# Dietary diversity and life satisfaction as mediators in the relationship between oral health and depression among older adults in china: a cross-sectional study

**DOI:** 10.3389/fpsyg.2025.1668797

**Published:** 2025-10-16

**Authors:** Keying Zhang, Yu Lei, Jiaqing Li, Shuaihao Zhu, Shuhui Sun

**Affiliations:** School of Management, Shandong Second Medical University, Weifang, China

**Keywords:** older adults, oral health, dietary diversity, life satisfaction, depression levels

## Abstract

Previous studies have demonstrated the relationship between oral health and depression in older adults. However, the specific effects and mechanisms of treatment require further investigation. Therefore, this study explored the mediating roles of dietary diversity and life satisfaction in the relationship between oral health and depression among older adults. Using data from the 2018 China Longitudinal Health and Longevity Study (CLHLS), the study participants were older adults aged 60 years and above, with a final sample size of 10,010 individuals. The severity of depression in older adults was assessed using the 10-item Center for Epidemiologic Studies Depression Scale (CESD-10). A dietary diversity score was calculated based on the frequency of food intake. Pearson’s correlation analysis is used to examine the connections between dental health, dietary diversity, life satisfaction, and depression levels. The Process 4.1 plugin model 6 in SPSS was used to analyze the mediating role of dietary diversity and life satisfaction in the relationship between oral health and depression levels. The frequency of tooth brushing, denture use, and the number of remaining teeth had statistically significant total effects on depression levels among older adults. Dietary diversity had a statistically significant direct effect on depression levels in relation to the number of remaining teeth. Dietary diversity and life satisfaction mediated the relationship between denture use and depression levels, as well as the relationship between brushing frequency and depression levels. Oral health improves and indirectly influences depression levels through dietary diversity and life satisfaction.

## 1 Introduction

Currently, human society is undergoing a global demographic aging trend, and this “silent revolution” has significantly impacted China. China is a “moderately aged” society and beginning to transition toward a “severely aged” society. By the end of 2024, China’s population aged 60 years or older exceeded 310 million, accounting for 22.0% of the total population, whereas those aged 65 years or older reached 220 million, representing 15.6% of the total population (Xiang and Xing, 2025). Older adults are highly prone to sickness and other negative life events, with their psychological trauma being more complex and intense. One of the main causes of impairment is depression (Wang et al., 2024), a mental illness marked by ongoing melancholy and disinterest (Vos et al., 2020). Studies conducted recently have revealed that over 35% of China’s older adults show indications of depression (Ni et al., 2017), bringing about considerable health and economic burdens for families and society alike (Usher et al., 2020). As people grow older, modifications in their social and familial obligations emerge, including retirement, transformations in leisure activities, and the experience of widowhood. These changes can exacerbate negative emotions and increase the risk of depression (Wang et al., 2022).

Considering the limited effectiveness of medications in treating depressive symptoms, research has focused on modifiable risk factors for depression. The connection between dental health and depression has drawn growing interest. Inadequate dental health may be a risk factor for prevalent systemic illnesses among older adults (Yun and Lee, 2020). Research indicates that oral health issues are associated with elevated levels of inflammatory markers, including serum C-reactive protein and white blood cell counts. Inflammatory markers can induce oxidative stress, which causes various mental disorders (Machado et al., 2021). Therefore, oral health directly influences depression among older adults. Oral ailments are a key public health problem in research on the elements affecting depressive signs among older adults. Brushing teeth is an important oral hygiene behavior that maintains oral hygiene and promotes oral health (Penmetsa et al., 2019). Poor oral hygiene can significantly increase the risk of depression among community-dwelling older adults (Zhang et al., 2022). Poor oral health is associated with unhealthy lifestyles, such as social withdrawal, isolation, and low self-esteem, which create a vicious cycle of deteriorating oral and mental health (Kenny et al., 2020). A cross-sectional study indicated that a low frequency of brushing teeth was associated with clinically significant depressive symptoms (Yuen et al., 2013).

Tooth loss is a major risk factor for depression (Wan et al., 2021), whereas regular tooth brushing can help prevent it. Structural abnormalities, such as tooth loss, cavities, or periodontal disease, as well as functional abnormalities, including dry mouth, ulcers, or cancerous changes, directly lead to reduced chewing efficiency, swallowing disorders, and a range of issues, including diminished taste perception and pain when eating. These physiological changes alter the sensory experience of food among older adults, significantly affecting their dietary preferences and food choices. A significant correlation exists between oral health status and dietary nutrient intake among older adults. Clinical studies have shown that oral health issues cause older adults to actively avoid foods that require thorough chewing, such as fiber-rich whole grains, fresh fruits, and vegetables, and opt for high-sugar and -fat processed foods. This change in dietary patterns ultimately leads to imbalances in nutrient intake (Liu and Huang, 2025), thereby affecting the overall health of older adults, potentially contributing to mental health issues such as depression. Dietary diversity is widely recognized as a crucial aspect of high-quality diets (Rouen and Wallace, 2017). The dietary diversity score (DDS) is a simple count of food groups consumed, consistent with dietary guideline recommendations, and serves as a global indicator of nutritional adequacy. Following the adjustment of socioeconomic and lifestyle factors, we found that a balanced and varied diet enhanced life satisfaction and alleviated depressive symptoms in older adults (Sanchez-Villegas et al., 2018). Low dietary quality diminishes contentment with food-related areas of life, and contentment with food-related areas of life is positively associated with overall life satisfaction (Ismael and Ploeger, 2020; Liu and Grunert, 2020; Oliveira et al., 2021). For example, marine omega-3 (n-3) fatty acids regulate dopaminergic and serotonergic neurotransmission, thereby reducing the risk of depression (Taylor and Holscher, 2020). A systematic review and meta-analysis of prospective cohort studies found that adherence to healthy dietary patterns, such as healthy/cautious, Mediterranean, plant-based (i.e., diets rich in plant-based foods relative to animal-based foods), and Tuscan diets, was associated with a 23% decrease in the risk of depression (Kris-Etherton et al., 2021). Life satisfaction constitutes a cognitive part of subjective well-being and is described as “a person’s general approval or positive viewpoint regarding their life” (An et al., 2023). Previous studies have shown that life satisfaction declines with age and that lower life satisfaction is associated with higher levels of depression (Hoseini-Esfidarjani et al., 2022). Sulandari et al. found that older adults with lower depression levels had approximately five times higher satisfaction than those with higher depression levels (Sulandari et al., 2024). Late adulthood is often associated with adverse life changes, leading to feelings of uselessness, loneliness, helplessness, dissatisfaction, and social withdrawal, among other symptoms (Van Damme-Ostapowicz et al., 2021). Wearing dentures to restore missing teeth in the anterior region (such as incisors, canines, and premolars) can improve facial aesthetics, alleviate inferiority in older adults, increase participation in social activities, enhance life satisfaction, and reduce the likelihood of depression (Huraib et al., 2022). Regular dental care for older adults can help maintain the health and function of their natural teeth for a long time or provide restorations, such as implants or dentures, when needed. This approach can effectively improve their life satisfaction and reduce depression (Finlayson et al., 2025). Therefore, oral health may serve as a predictive indicator of healthy aging, including well-being, satisfaction, and a sense of accomplishment, as advocated by the World Health Organization (Gao et al., 2020).

According to the biopsychosocial model, an individual’s physical and mental health is closely related to and influenced by biological, psychological, and social factors that interact with one another (Almeida et al., 2024). The deterioration of oral health among the elderly reduces dietary diversity and leads to social withdrawal due to embarrassment, fostering feelings of loneliness, lowering life satisfaction, and resulting in depression. Existing studies on depression among older adults have validated the interrelationships among oral health, dietary diversity, and life satisfaction. However, research on the relationships among the four ariables is insufficient. Therefore, this study explored the impact of oral health on depression levels among older adults and the mediating effects of dietary diversity and life satisfaction.

## 2 Methods

### 2.1 Data sources

**Figure 1:**
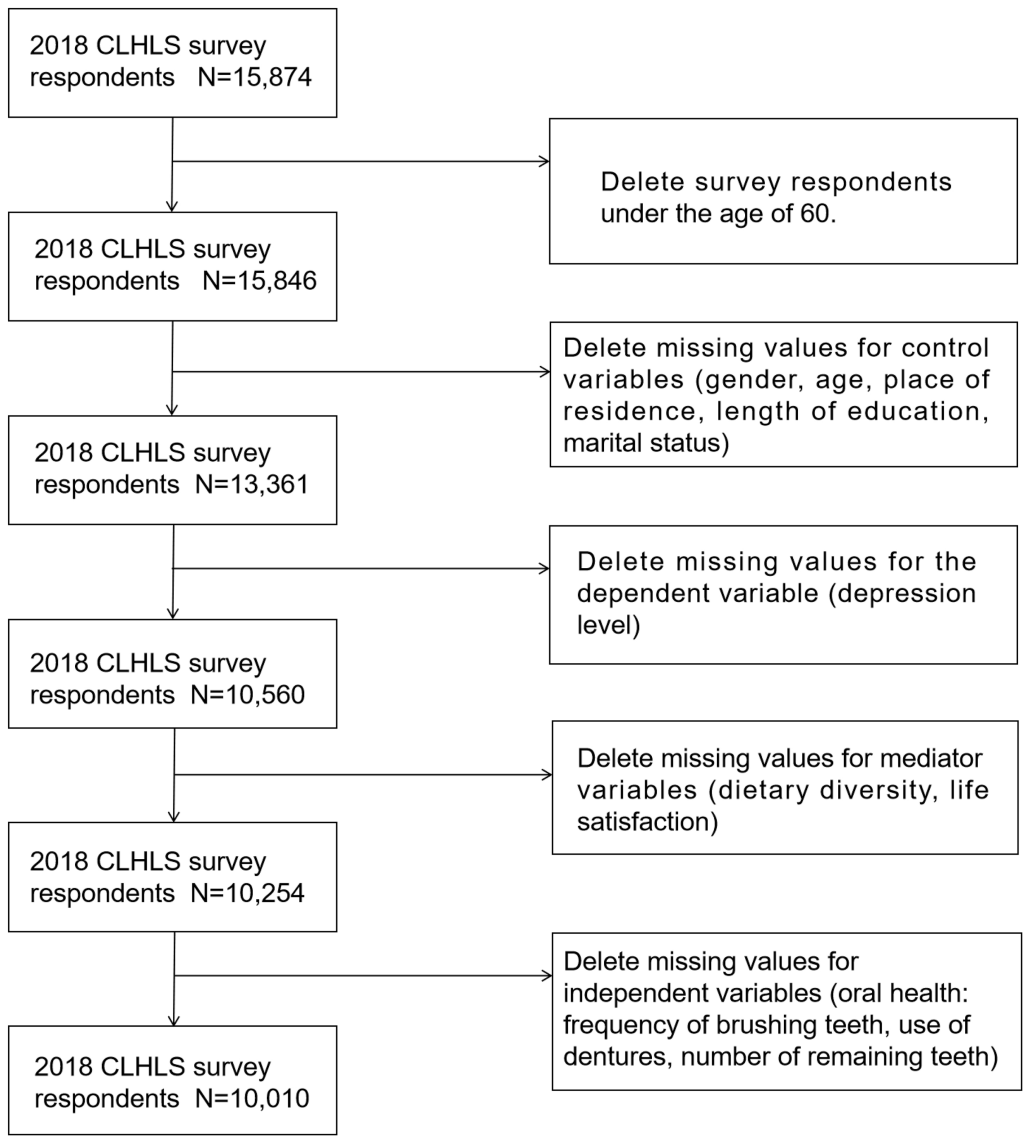
Flow chart of sample inclusion.

The study population and data sources were derived from the 2018 China Long-term Health and Longevity Survey (CLHLS) conducted by the Center for Healthy Aging and Development at Peking University. The CLHLS is a comprehensive national study of older adults in China. It used a multistage disproportionately targeted random sampling method. The survey covered 23 provinces, municipalities, and autonomous regions, encompassing approximately 85% of the Chinese population. The CLHLS received approval from the ethics committees of Peking University and Duke University and was carried out in line with the authorized guidelines. The inclusion criteria for the study participants were as follows: age ≥60 years and no missing values for core variables. Ultimately, 10,010 participants were included in this study. The screening process is illustrated in Figure 1.

### 2.2 Measures

#### 2.2.1 Oral health

Oral health encompasses denture usage, oral hygiene practices, and the count of natural teeth. Denture usage was ascertained by inquiring whether older adults wore dentures, with a value of one assigned to a yes response and zero to a no response. The oral hygiene practice indicator was defined as the daily frequency of tooth brushing among older adults. The brushing frequency was categorized into 0, 1, 2, 3, and 4, corresponding to “never,” “occasionally,” “once a day,” “twice a day,” and “three or more times a day,” respectively (Lou et al., 2024). The number of natural teeth is gauged based on answers to the question “How many natural teeth do you have (excluding dentures)?” with a scoring range of 0–36 points (Yang et al., 2023). Higher scores signified a greater number of teeth in older adults.

#### 2.2.2 Mediating variables

##### 2.2.2.1 Dietary diversity

Dietary diversity is a crucial aspect of health and survival (Xian et al., 2024). The more monotonous a person’s diet, the more limited their nutrient intake. Foods with monotonous flavors lack balanced nutrients and cannot meet the nutritional needs of the body. Therefore, this study used the DDS to assess the nutritional status (Zhu et al., 2024), which included nine categories: fruits, vegetables, meat, fish, eggs, tofu products, dairy products, nuts, and tea consumption. Among these, fruits and vegetables had four options: those who answered “daily, almost daily,” and “often” earned one point, whereas those who answered “sometimes,” “rarely,” or “never” earned zero points. The consumption frequency of meat, fish, eggs, soy products, dairy products, nuts, and tea was assessed using five options. Respondents who answered “almost daily,” “not daily,” and “at least once a week” earned one point, whereas those who answered “not weekly,” “at least once a month,” “not monthly,” “occasionally,” “rarely,” and “never” earned zero points. The results were calculated by summing all scores, with a score range of 0–9. Higher scores indicated adequate health. We conducted a factor analysis suitability test on the data, yielding a KMO value of 0.749 and a significant Bartlett’s sphericity test (χ^2^ = 7478.69, *p* < 0.001), indicating suitability for factor analysis. Reliability analysis was also performed, yielding a Cronbach’s alpha reliability coefficient of 0.637, indicating reasonable reliability.

##### 2.2.2.2 Life satisfaction

The CLHLS assessed the life satisfaction of older adults with the question (Zhu et al., 2023), “How is your life now?” Responses were scored from 5 (very good) to 1 (very poor), with scores ranging from 1 to 5. The higher the score, the higher the participant’s life satisfaction.

#### 2.2.3 Level of depression

Referring to previous literature (Lou et al., 2024), the CESD-10 depression scale from the CLHLS was used to assess depression. The scale included three questions related to positive emotions: “Are you hopeful regarding the future?” and “Are you as happy as you were when you were younger?” “How is your sleep quality now?” The response options were “*always*,” “*often*,” “*sometimes*,” “*rarely*,” and “*never*,” with scores ranging from 0 to 5. Seven questions represented negative emotions, such as “Do you get upset over delicate matters?” Do you find it difficult to concentrate on your activities?” “Are you sad or depressed?” and “Do you believe that as you get older, you become less capable and find it difficult to carry out activities?” These questions were reverse-scored, with a total score range of 0–50 points. Higher scores indicated a higher depression level. Cronbach’s alpha reliability coefficient was 0.807, indicating excellent reliability and sound design in this study.

#### 2.2.4 Demographic characteristics

This study selected five factors that may affect depression among older adults as control variables, such as the continuous variables of age and years of education, as well as the categorical variables of gender, place of residence, and marital status.

### 2.3 Statistical analysis

This study is a descriptive correlational study, aiming to examine the relationship between oral health, dietary diversity, life satisfaction, and depression levels among older adults. Data analysis was performed using SPSS version 25.0. Statistical descriptions of the demographic characteristics and main variables were obtained. Variables are presented as mean ± standard deviation and frequency. *T*-tests and analysis of variance (ANOVA) were employed to compare the discrepancies in depression levels across the groups. Pearson’s correlation analysis measured the bivariate correlations among oral health, dietary diversity, life satisfaction, and depression levels. This study employed Hayes’ PROCESS macro (SPSS software version 4.1, model 6) to examine the chained mediation model. Oral health among the elderly served as the independent variable, depression levels as the dependent variable, dietary diversity as the first mediating variable, and life satisfaction as the second mediating variable. A 95% confidence interval adjusted for sampling bias was generated through 5,000 bootstrap samples, and effect sizes were estimated using standardized coefficients.

## 3 Results

### 3.1 Baseline characteristics of the target group

The study included 10,010 older individuals with an average age of 83.12 ± 11.32 years, including 4,553 men and 5,457 women; 2,562 and 7,448 lived in urban and rural areas, respectively; 4,713 were married 47.1% and 5,297 were unmarried 52.9%. The average tooth brushing frequency was 1.91 ± 1.12 points, the average number of natural teeth was 10.79 ± 10.68 points, and the average DDS was 4.78 ± 2.02 points. Significant differences were observed between sex, age, place of residence, years of education, marital status, and depression levels (see Table 1).

**Table 1:** Descriptive statistics.

Variables	All	Depression level score	*t*	*p*-value
Age	83.12 ± 11.32			
Sex			−10.624	<0.001
Male	4,553 (45.5)	21.47 ± 5.99		
Female	5,457 (54.5)	22.77 ± 6.18		
Residence			25.524	<0.001
Urban	2,562 (25.6)	21.46 ± 6.20		
Suburban	3,275 (32.7)	22.57 ± 6.14		
Rural	4,173 (41.7)	22.31 ± 6.03		
Marital status			−12.035	<0.001
Married	4,713 (47.1)	21.41 ± 5.94		
Unmarried	5,297 (52.9)	22.87 ± 6.20		
Years of education	3.74 ± 4.46			
Toothbrushing frequency score	1.91 ± 1.12			
Denture use score	0.40 ± 0.50			
Remaining teeth score	10.79 ± 10.68			
Dietary diversity score	4.78 ± 2.03			
Life satisfaction score	3.92 ± 0.80			

### 3.2 Correlation analysis

Analysis results indicate that brushing frequency is positively correlated with dietary diversity and life satisfaction, while negatively correlated with depression levels among older adults. Similarly, denture use is positively correlated with dietary diversity and life satisfaction, and negatively correlated with depression levels among older adults. The number of remaining teeth was positively correlated with dietary diversity and life satisfaction, and negatively correlated with depression levels among the elderly. Furthermore, dietary diversity was positively correlated with life satisfaction among the elderly and negatively correlated with their depression levels. Life satisfaction was also negatively correlated with depression levels among the elderly (see Table 2).

**Table 2:** Pearson correlation analysis (r).

Variables	Brushing frequency	Denture use	Remaining teeth	Dietary diversity	Life satisfaction	Depression level
Frequency of brushing teeth	1.000					
Use of dentures	0.254^**^	1.000				
Remaining teeth	0.192^**^	−0.310^**^	1.000			
Dietary diversity	0.284^**^	0.108^**^	0.153^**^	1.000		
Life satisfaction	0.093^**^	0.086^**^	0.024^*^	0.207^**^	1.000	
Level of depression	−0.119^**^	−0.082^**^	−0.090^**^	−0.197^**^	−0.413^**^	1.000

***indicates *p* < 0.001, **indicates *p* < 0.01, and *indicates *p* < 0.05.

### 3.3 Analysis of mediating effects

This study used descriptive and correlation analyses to construct a chain mediation model, in which oral health served as the independent variable, depression levels as the dependent variable, and dietary diversity and life satisfaction as sequential mediators. The analysis controlled for potential confounders, including age, sex, marital status, and years of education.

**Figure 2:**
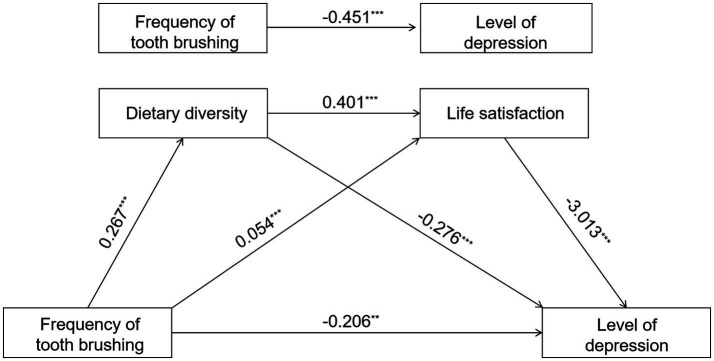
Diagram of chain mediating effect model. ***p < 0.001, **p < 0.01, and *p < 0.05.

As shown in the figure, all path coefficients are statistically significant. Specifically, reduced toothbrushing frequency among older adults is significantly associated with decreased dietary diversity (*β* = 0.267, *p* < 0.001) and lower life satisfaction (*β* = 0.054, *p* < 0.001). Conversely, reduced dietary diversity not only predicted decreased life satisfaction (*β* = 0.401, *p* < 0.001) but also foreshadowed elevated depression levels (*β* = −0.276, *p* < 0.001). Furthermore, decreased life satisfaction served as a potent predictor of increased depression levels (*β* = −3.013, *p* < 0.001). After controlling for mediating variables, the direct effect of brushing frequency on depression remained statistically significant (*β* = −0.206, *p* < 0.001). Model 3 explains a significant proportion of the variance in depression levels (*R*^2^ = 0.203) (Table 3 and Figure 2).

**Table 3:** Results of chain mediation effect regression analysis of toothbrushing frequency.

Variables	Model 1				Model 2				Model 3			
	Life satisfaction				Dietary diversity				Level of depression			
	Coeff	*Se*	*t*	*P*	Coeff	*Se*	*t*	*P*	Coeff	*Se*	*t*	*P*
Constant	3.469	0.085	40.803	<0.001	3.266	0.206	15.794	<0.001	32.079	0.642	49.910	<0.001
Sex	0.027	0.017	1.543	0.122	−0.127	0.039	−3.235	<0.01	0.825	0.121	6.813	<0.001
Age	0.005	0.001	6.394	<0.001	0.006	0.002	3.293	<0.01	0.028	0.006	4.745	<0.001
Place of residence	−0.048	0.010	−4.419	<0.001	−0.510	0.024	−20.738	<0.001	−0.299	0.077	−3.884	<0.01
Years of education	0.009	0.002	4.203	<0.001	0.100	0.005	20.295	<0.001	−0.037	0.015	−2.442	<0.05
Marital status	−0.055	0.196	−2.820	<0.01	−0.117	0.044	−2.656	<0.01	0.509	0.135	3.752	<0.01
Frequency of brushing teeth	0.054	0.007	7.093	<0.001	0.267	0.017	15.330	<0.001	−0.206	0.054	−3.800	<0.01
Life satisfaction					0.401	0.022	17.806	<0.001	−3.013	0.070	−42.911	<0.001
Dietary diversity									−0.276	0.030	−9.015	<0.001
R-sq	0.017				0.227				0.203			

**Figure 3:**
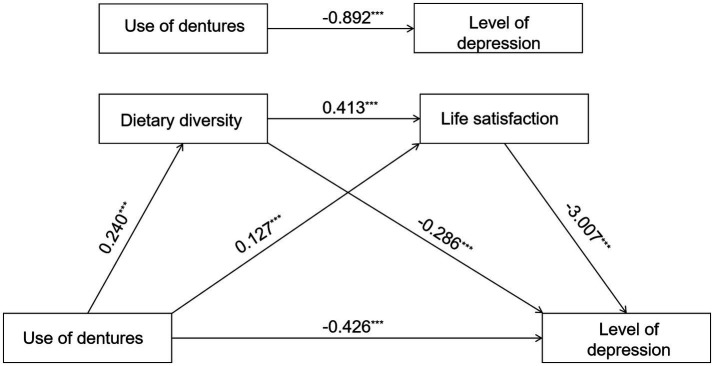
Diagram of chain mediating effect model. ***p < 0.001, **p < 0.01, and *p < 0.05.

Similarly, as shown in the figure, all path coefficients are statistically significant. Specifically, denture use among older adults positively predicts dietary diversity (*β* = 0.240, *p* < 0.001) and life satisfaction (*β* = 0.127, *p* < 0.001). Dietary diversity positively predicted life satisfaction (*β* = 0.413, *p* < 0.001) and negatively predicted depression levels (*β* = −0.286, *p* < 0.001). Life satisfaction negatively predicted depression levels (*β* = −3.007, *p* < 0.001). After controlling for mediating variables, the direct effect of denture use on depression remained statistically significant (*β* = −0.426, *p* < 0.001). Model 3 explains a significant proportion of the variance in depression levels (*R*^2^ = 0.203) (Table 4 and Figure 3).

**Table 4:** Results of chain mediation effect regression analysis for denture use.

Variables	Model 1				Model 2				Model 3			
	Life satisfaction				Dietary diversity				Level of depression			
	Coeff	*Se*	*t*	*P*	Coeff	*Se*	*t*	*P*	Coeff	*Se*	*t*	*P*
Constant	3.594	0.081	44.381	<0.001	4.030	0.201	19.988	<0.001	31.616	0.625	50.568	<0.001
Sex	0.037	0.017	2.173	<0.05	−0.072	0.039	−1.838	0.066	0.783	0.120	6.497	<0.001
Age	0.004	0.001	5.384	<0.001	0.001	0.002	0.879	0.379	0.032	0.005	5.407	<0.001
Place of residence	−0.058	0.010	−5.413	<0.001	−0.572	0.024	−23.411	<0.001	−0.266	0.076	−3.492	<0.001
Years of education	0.011	0.002	5.443	<0.001	0.114	0.004	23.251	<0.001	−0.046	0.015	−3.022	<0.01
Marital status	−0.054	0.019	−2.798	<0.01	−0.122	0.044	−2.751	<0.01	0.507	0.135	3.739	<0.001
Denture use	0.127	0.016	7.846	<0.001	0.240	0.036	6.516	<0.001	−0.426	0.112	−3.789	<0.001
Life satisfaction					0.413	0.022	18.192	<0.001	−3.007	0.070	−42.771	<0.001
Dietary diversity									−0.286	0.030	−9.436	<0.001
R-sq	0.019				0.212				0.203			

**Figure 4:**
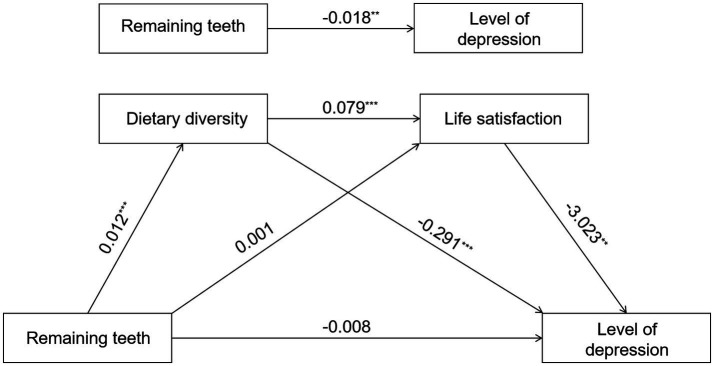
Diagram of chain mediating effect model. ***p < 0.001, **p < 0.01, and *p < 0.05.

Research findings indicate that the number of remaining teeth in older adults positively correlates with dietary diversity (*β* = 0.012, *P* < 0.001). Dietary diversity, in turn, positively correlates with life satisfaction (*β* = 0.079, *P* < 0.001) and negatively correlates with depression levels (*β* = −0.291, *P* < 0.001). Life satisfaction also showed a negative correlation with depression levels (*β* = −3.023, *p* < 0.001). Model 3 explains a significant proportion of the variance in depression levels (*R*^2^ = 0.202) (Table 5 and Figure 4).

**Table 5:** Results of chain mediation regression analysis for remaining teeth.

Variables	Model 1				Model 2				Model 3			
	Dietary diversity				Life satisfaction				Level of depression			
	Coeff	*Se*	*t*	*P*	Coeff	*Se*	*t*	*P*	Coeff	*Se*	*t*	*P*
Constant	5.101	0.208	24.461	<0.001	3.158	0.091	34.553	<0.001	31.888	0.679	46.954	<0.001
Sex	−0.054	0.040	−1.356	0.175	0.043	0.017	2.508	<0.05	0.780	0.120	6.471	<0.001
Age	0.008	0.002	4.001	<0.001	0.004	0.001	5.201	<0.001	0.028	0.006	4.453	<0.001
Place of residence	−0.598	0.024	−24.076	<0.001	−0.014	0.010	−1.284	0.199	−0.260	0.076	−3.413	<0.01
Years of education	0.116	0.005	23.197	<0.001	0.002	0.002	1.092	0.274	−0.044	0.015	−2.898	<0.01
Marital status	−0.146	0.045	−3.224	<0.01	−0.045	0.019	−2.332	<0.05	0.510	0.135	3.764	<0.01
Remaining teeth	0.012	0.002	6.097	<0.001	0.001	0.001	1.320	0.186	−0.008	0.006	−1.454	0.145
Dietary diversity					0.079	0.004	18.606	<0.001	−0.291	0.030	−9.598	<0.001
Life satisfaction									−3.023	0.070	−43.060	<0.001
R-sq	0.184				0.046				0.202			

The overall effect of brushing frequency on depression levels was significant (*β* = −0.451, 95% CI = [−0.567 to −0.336]), with both the direct effect (*β* = −0.206, 95% CI = [−0.312 to −0.099]) and the total indirect effect (*β* = −0.245, 95% CI = [−0.297 to −0.193]). Within the indirect pathway, both dietary diversity (*β* = −0.165, 95% CI = [−0.212 to −0.119]) and life satisfaction (*β* = −0.074, 95% CI = [−0.093 to −0.055]) were significant independent mediators, and the chained mediating effect formed by the two was also significant (*β* = −0.006, 95% CI = [−0.008 to −0.004]). The overall effect of denture use on depression levels was strongest (*β* = −0.892, 95% CI = [−1.134 to −0.650]), with its direct effect (*β* = −0.426, 95% CI = [−0.647 to −0.205]), and the total indirect effect (*β* = −0.466, 95% CI = [−0.571 to −0.358]). Dietary diversity served as the primary mediating pathway (*β* = −0.381, 95% CI = [−0.482 to −0.283]), followed by the independent mediation of life satisfaction (*β* = −0.069, 95% CI = [−0.095 to −0.045]), and the chained mediation involving both (*β* = −0.015, 95% CI = [−0.020 to −0.010]) were also significant. The overall effect of remaining teeth on depression levels was relatively small but significant (*β* = −0.018, 95% CI = [−0.031 to −0.005]), primarily mediated through indirect pathways (*β* = −0.009, 95% CI = [−0.015 to −0.004]), where dietary diversity (*β* = −0.003, 95% CI = [−0.005 to −0.002]) and the chained mediating path “dietary diversity → life satisfaction” (*β* = −0.002, 95% CI = [−0.003 to −0.002]) made significant contributions (see Table 6).

**Table 6:** Mediation effect test.

Pathway	Effect (95%CI)	$S_{\bar{x}}$
Frequency of brushing teeth		
Total effect	−0.451 (−0.567 ~ −0.336)	0.058
Direct effect	−0.206 (−0.312 ~ −0.099)	0.054
Total indirect effect	−0.245 (−0.297 ~ −0.193)	0.026
Frequency of brushing teeth→dietary diversity → level of depression	−0.165 (−0.212 ~ −0.119)	0.023
Frequency of brushing teeth→life satisfaction→ level of depression	−0.074 (−0.093 ~ −0.055)	0.009
Frequency of brushing teeth →dietary diversity→life satisfaction→ level of depression	−0.006 (−0.008 ~ −0.004)	0.001
Denture use		
Total effect	−0.892 (−1.134 ~ −0.650)	0.123
Direct effect	−0.426 (−0.647 ~ −0.205)	0.112
Total indirect effect	−0.466 (−0.571 ~ −0.358)	0.053
Denture use**→**dietary diversity → level of depression	−0.381 (−0.482 ~ −0.283)	0.050
Denture use**→**life satisfaction→ level of depression	−0.069 (−0.095 ~ −0.045)	0.013
Denture use**→**dietary diversity→life satisfaction→ level of depression	−0.015 (−0.020 ~ −0.010)	0.002
Remaining teeth		
Total effect	−0.018 (−0.031 ~ −0.005)	0.006
Direct effect	−0.008 (−0.020 ~ 0.003)	0.006
Total indirect effect	−0.009 (−0.015 ~ −0.004)	0.002
Remaining teeth→dietary diversity→level of depression	−0.003 (−0.005 ~ −0.002)	0.001
Remaining teeth→life satisfaction→level of depression	−0.003 (−0.008 ~ 0.001)	0.013
Remaining teeth→dietary diversity→life satisfaction→level of depression	−0.002 (−0.003 ~ −0.002)	0.001

## 4 Discussion

### 4.1 Relationship between oral health and the level of depression

This study indicated that brushing frequency negatively forecasted depression levels in older adults, consistent with the findings of Zhang et al. (2022). Poor oral health can impair social functioning, potentially leading to reduced participation in social activities. For example, individuals who have lost their teeth may encounter difficulties eating, speaking, smiling, or showing their teeth, which may lead them to eat alone. Eating alone is a risk factor for depression in older adults (Yamamoto et al., 2017). Oral hygiene positively affects health (Coll et al., 2020). Chronic inflammation caused by oral infections such as periodontitis can lead to changes in hormone and neurotransmitter levels in the brain, thereby contributing to depression (Palomer et al., 2024). Regular brushing helps maintain good hygiene and eliminate harmful bacteria, contributing to good oral health and reducing the likelihood of depression among the older adult population. Interventions such as increasing the knowledge and awareness of oral health can help older adults protect their natural teeth. Additional investigation has shown that denture usage is negatively associated with depression in older adults. After removing the remaining roots and crown fragments and fitting a suitable denture, the chronic pain in the mouth was eliminated, directly improving the patient’s mood (Cross et al., 2025). Compared with older adults with 20 or more natural teeth, those with zero to nine teeth who did not wear dentures exhibited severe depressive symptoms. In contrast, those with zero to nine teeth who wore dentures had a relatively lower risk of depression (Nakazawa et al., 2024). Communities should intensify outreach efforts to educate seniors and their families about the close connection between oral health and mental well-being. They should encourage seniors to undergo regular dental checkups and improve their daily oral hygiene practices.

### 4.2 Mediating effects of dietary diversity and life satisfaction

Research findings indicated that remaining teeth can influence depression levels among older adults through dietary diversity. Oral health issues are a major determinant of malnutrition in older adults, and malnutrition is associated with depression in later life (Hwang et al., 2022). Malnutrition and insufficient energy intake directly impact brain health and neurotransmitter synthesis, physiologically increasing susceptibility to depression (Ahmadi-Khorram et al., 2025). Proper nutrition is vital for sustaining physical health and guaranteeing normal brain operation. A greater number of natural teeth facilitates biting and chewing. In contrast, fewer natural teeth may lead to difficulty chewing hard or crisp foods, resulting in avoidance of certain foods and an increased likelihood of choosing softer, energy-dense foods (saturated fats and sugars) (Zhao et al., 2021). Consistent toothbrushing is an advantageous hygiene practice that eliminates harmful bacteria from the mouth. This aids in maintaining sound oral health and raises the probability of older adults consuming a variety of foods, thus lowering the risk of depression. This is consistent with Sun’s research findings (Sun et al., 2022). Studies have identified various health risks associated with tooth loss (Folayan et al., 2021). Dealing with this problem via dental restoration can boost chewing, thereby permitting older adults to absorb necessary nutrients effectively. When investigating simple nutritional assessments, denture use increased the simple nutritional scores. Compared with edentulous patients, healthier dietary patterns were associated with higher rates of good nutrition (Lou et al., 2024). A high consumption of fruits, vegetables, fish, and whole grains marks a wholesome diet. Undernutrition may result in physical debility and tiredness, thereby raising the chance of developing depression (Huang et al., 2025). For example, iron is crucial in hemoglobin synthesis, and iron deficiency anemia could cause insufficient oxygen delivery to the brain, thereby affecting the nervous system function, leading to symptoms such as fatigue, difficulty concentrating, mood swings, and depression (Huang et al., 2025). Fresh vegetables act as an ample provider of essential nutrients such as proteins, vitamins (notably vitamins C and B), minerals (encompassing potassium, calcium, and magnesium), dietary fiber, and a range of antioxidants like carotenoids and polyphenols—all of which are crucial for human well-being. These nutrients play a key role in sustaining the mental health of older adults and lessening their tendency to suffer from depression, anxiety, and dementia (Tomita et al., 2020). Communities can organize free dental clinics to provide discounted treatment services for the elderly. Introducing soft-food nutritional meal options at community senior dining halls not only facilitates chewing but also ensures adequate nutrition, thereby alleviating symptoms of depression among older adults.

Ensuring excellent oral hygiene is crucial for portraying older individuals as healthy and enhancing their lifespan and overall well-being (Marya et al., 2020). Individuals with poor oral health who have difficulty chewing or communicating verbally and emotionally may experience a decline in self-confidence, rendering them more susceptible to loneliness and reducing their life satisfaction. This is consistent with the research of Badewy et al. (2021). Studies have revealed that 20% of Chinese adults aged 65 and above seldom or never brush their teeth, while 38% brush their teeth twice or more each day. Older adults who rarely or never brush their teeth are significantly more prone to depression, while those who brush their teeth twice or more a day have a relatively lower risk of depression. This discrepancy is because older people who neglect oral hygiene are susceptible to oral inflammation, which affects their eating habits, quality of life, and mental well-being, potentially resulting in higher rates of depression. For instance, compared to those with good oral health, people with periodontitis experience a marked drop in their quality of life and life satisfaction, which in turn further impacts their mental health and emotional condition (Huang et al., 2025). Neuroinflammation caused by periodontitis-related systemic inflammation or periodontal pathogens and their inflammatory products directly invading the brain is associated with the onset and progression of depression. Older individuals who do not wear dentures or have ill-fitting dentures often have a more restricted diet, unhealthy, and, consequently, or it may directly lower overall life satisfaction due to negative evaluations of one’s current circumstances, rendering them more prone to depression than those using full dentures (Satishkumar et al., 2021). The use of dentures can improve chewing difficulties, oral articulation issues, and facial aesthetics caused by tooth loss, thereby promoting nutritional and social well-being and enhancing overall life satisfaction (Skomina et al., 2025). Research indicates that retaining more teeth and using dentures are independently associated with happiness. Older individuals with fewer teeth experience a relatively slight decline in happiness after denture use (Abbas et al., 2024). Family members should assist older adults in promptly undergoing denture restoration and dental treatment to restore basic functions. They should also encourage older adults to utilize cognitive behavioral therapy and other approaches to help them reconstruct their perceptions of aging and self-identity, thereby enhancing life satisfaction and reducing the likelihood of depression.

### 4.3 Analysis of chain mediation effects

Research indicates that the number of remaining teeth directly influences depression among older adults and can indirectly affect depression through dietary diversity and life satisfaction. Losing teeth can impair one’s ability to chew and swallow, and reduced chewing capability is a significant oral factor that can lead to depression. Difficulty chewing often stems from oral infections such as periodontitis, which release pro-inflammatory cytokines like IL-6 and TNF-*α*. This chronic, low-grade systemic inflammation can impair brain function by inhibiting serotonin synthesis and activating the stress response system (HPA axis)—a core physiological mechanism underlying depression (Cao et al., 2025). Older individuals with chewing difficulties might prefer foods that are easier to chew, potentially overlooking more nutritionally balanced options. This can result in nutrient deficiencies and an increased risk of malnutrition. Malnutrition is a risk factor for various chronic diseases, exacerbating older adults’ concerns regarding their health status and emotional distress, and further intensifying their depressive tendencies (Bozkurt et al., 2024). For example, reduced meat intake leads to muscle loss and weakness, with fatigue being a core diagnostic criterion for depression.

Additionally, reduced chewing ability may affect older adults’ enjoyment of food, thereby affecting their positive eating experiences, causing psychological discomfort, lowering life satisfaction, and depressive symptoms (Folayan et al., 2021). Difficulties in swallowing affect health-related quality of life, whereas low quality of life satisfaction is associated with depression (Maki et al., 2024). Dental implants eliminate sleep disturbances, irritability, and low mood caused by persistent toothaches. Their superior stability alleviates the significant fear and embarrassment experienced by removable denture wearers, reducing the psychological burden of tooth loss and effectively alleviating depressive symptoms in the elderly (Davangere et al., 2025). Research has Shown that tooth brushing can improve oral health, particularly by reducing the risk of depression in men (Zhao et al., 2022). Adequate oral health contributes to quality of life. Satisfactory chewing efficiency, clean, aesthetically pleasing dentition, and dentures can enhance the quality of life in terms of self-esteem and personal image (Gao et al., 2020). Research into dental and oral issues among older adults and their behavior when seeking treatment has shown that adequate access to medical and dental services can reduce the risk of premature illness and mortality, preserve functionality, and improve overall quality of life.

### 4.4 Implications

This study focuses on examining the mediating role of dietary diversity and life satisfaction in the relationship between oral health and depression levels among the elderly population in China. Based on cross-sectional survey data, we hypothesize and validate a dual mediation model: On one hand, good oral health function helps maintain a diverse dietary pattern, and adequate nutritional intake has been shown to positively influence emotional regulation. On the other hand, oral health positively impacts psychological well-being by enhancing overall life satisfaction, ultimately reducing depression risk. The findings reveal key pathways linking dietary and psychological factors in the association between oral health and mental well-being, providing important theoretical foundations and practical implications for developing comprehensive intervention strategies aimed at improving the physical and mental health of older adults in China.

### 4.5 Limitations

This study has some limitations. The cross-sectional nature of the data precludes establishing definitive causal relationships. Future research should address this limitation by conducting longitudinal studies with extended follow-up periods.

## 5 Conclusion

Older individuals should focus on maintaining a balanced diet and reducing intake of irritating substances to maintain excellent oral health. Communities can enhance oral hygiene awareness among older adults through lectures and educational videos, encouraging them to maintain regular and effective oral hygiene habits. Regular oral examinations are extremely important for older adults because they can help promptly detect and treat oral diseases, thereby improving their quality of life. In summary, this study found that oral health in older adults has a direct impact on depression and validated the chain-mediated role of dietary diversity and life satisfaction between oral health and depression. However, the variables and data used in this study may have certain limitations; therefore, further research on this topic is warranted.

## Data Availability

The datasets presented in this study can be found in online repositories. The names of the repository/repositories and accession number(s) can be found in the article/supplementary material.
